# A Free-Choice High-Fat High-Sugar Diet Alters Day–Night *Per2* Gene Expression in Reward-Related Brain Areas in Rats

**DOI:** 10.3389/fendo.2018.00154

**Published:** 2018-04-09

**Authors:** Aurea Susana Blancas-Velazquez, Unga A. Unmehopa, Leslie Eggels, Laura Koekkoek, Andries Kalsbeek, Jorge Mendoza, Susanne E. la Fleur

**Affiliations:** ^1^Institute of Cellular and Integrative Neurosciences, CNRS UPR-3212, University of Strasbourg, Strasbourg, France; ^2^Department of Endocrinology and Metabolism, Academic Medical Center, University of Amsterdam, Amsterdam, Netherlands; ^3^Laboratory of Endocrinology, Department of Clinical Chemistry, Academic Medical Center, University of Amsterdam, Amsterdam, Netherlands; ^4^Netherlands Institute of Neuroscience, Institute of the Royal Academy of Arts and Sciences, Amsterdam, Netherlands

**Keywords:** *Per2*, fat and sugar, clock-genes, obesity, reward, nucleus accumbens

## Abstract

Under normal light–dark conditions, nocturnal rodents consume most of their food during the dark period. Diets high in fat and sugar, however, may affect the day–night feeding rhythm resulting in a higher light phase intake. *In vitro* and *in vivo* studies showed that nutrients affect clock-gene expression. We therefore hypothesized that overconsuming fat and sugar alters clock-gene expression in brain structures important for feeding behavior. We determined the effects of a free-choice high-fat high-sugar (fcHFHS) diet on clock-gene expression in rat brain areas related to feeding and reward and compared them with chow-fed rats. Consuming a fcHFHS diet for 6 weeks disrupted day–night differences in *Per2* mRNA expression in the nucleus accumbens (NAc) and lateral hypothalamus but not in the suprachiasmatic nucleus, habenula, and ventral tegmental area. Furthermore, short-term sugar drinking, but not fat feeding, upregulates *Per2* mRNA expression in the NAc. The disruptions in day–night differences in NAc *Per2* gene expression were not accompanied by altered day–night differences in the mRNA expression of peptides related to food intake. We conclude that the fcHFHS diet and acute sugar drinking affect *Per2* gene expression in areas involved in food reward; however, this is not sufficient to alter the day–night pattern of food intake.

## Introduction

The suprachiasmatic nucleus (SCN) controls the circadian (24-h period) rhythms in behavior and physiology (1, 2). In the SCN and in all cells of the body, a feedback loop of genes (known as clock genes) are expressed and repressed with a 24-h period. The positive limb of the loop consists of the genes *Clock* and *Bmal1* of which the protein dimer promotes *Per* and *Cry* expression, and genes from the negative limb which protein products repress *Clock* and *Bmal1* activity (3). Environmental light is the main synchronizer for the SCN (4), whereas other brain circadian clocks are more sensitive to internal hormonal and metabolic signals. Thus, feeding cues are also able to modify the day/night physiological variation. Circadian eating patterns can be altered by high-energy diets (5–7) such as the free-choice high-fat high-sugar (fcHFHS) diet, consisting of the choice between tap water, chow-food, fat, and sugar (8). Rodents exposed to a fcHFHS diet show smaller day–night differences in food intake. Especially intake of fat and sugar components of the diet does not show day–night variations, whereas the intake of the nutritionally balanced chow diet remains rhythmic with a higher intake in the dark period when animals are active (8, 9). Moreover, we previously reported changes in the molecular clock properties of the lateral habenula (LHb) in fcHFHS diet-exposed mice, an area involved in reward-related behavior, whereas clock proteins in the arcuate nucleus, an important area for homeostatic feeding, were unchanged (9). It remains, however, to be determined whether molecular clock-gene expression in food-related reward circuitry, such as striatum and lateral hypothalamus (LH), are affected by a diet high in fat and sugar and if these effects are involved in disruption of the day/night feeding rhythm. We hypothesize that the obesogenic diet-induced disruption of day–night palatable intake is linked to nutrients (such as fat and sugar) affecting the brain oscillators within the food reward circuitry. In this study, we exposed rats to a fcHFHS diet for 6 weeks and measured clock-genes and food-related peptide gene expression in different reward-related brain areas. Subsequently, we evaluated the acute effects of sugar intake on *Per2* gene expression in the nucleus accumbens (NAc) of rats.

## Materials and Methods

Male Wistar rats weighing ~250 g were single-housed in Plexiglas cages in a temperature and light-controlled room with 21–23°C and a 12:12 h light:dark-cycle ZT0 at 7:00 a.m. (Zeitgeber Time: ZT0 onset of light and ZT12 when lights are off). Animals were fed with regular chow and water *ad libitum* during baseline. All experiments were approved by the Animal Ethics Committee of the Royal Netherlands Academy of Arts and Sciences (Amsterdam).

### fcHFHS-Diet Effects on Clock-Gene and Output-Genes Expression in Feeding-Related Areas

Rats were either fed chow (*n* = 14) or the fcHFHS diet (*n* = 14): tap water, chow-food, 30% sucrose-water bottle, and a dish with fat (beef tallow, Vandemoortele, Belgium). Food intake was measured 3 times/week and 1 time/week at the beginning and the end of the day and night phases to assess the day–night food intake. Body weight was measured at least twice/week. After 6 weeks, rats from both groups were divided and euthanized at two different time points: ZT4 (day point) and ZT16 (night point) by sedation in a CO_2_-chamber and immediately decapitated. Brains were quickly removed, frozen, and stored at −80°C. Epididymal and perirenal white adipose tissue (WAT) was dissected and weighted.

### Sugar Intake Effect on *Per2* mRNA Expression in NAc

Rats were divided into two groups. During 7 days at ZT 10 (2 h before lights off), one group received an extra bottle of water (*n* = 8) and the other group a bottle with 30% sugared water (*n* = 9) during 2.5 min to consume ~5 kcal of sugar. To determine exact sugar intake, the bottle was weighted before and after drinking. Rats were sacrificed 30 min after last water or sugar intake. Animals were sedated and decapitated and brains were harvested as described above.

### mRNA Extraction and Quantitative Real-Time PCR

Punches from frozen brains were taken using a small needle dissecting NAc, SCN, LH, habenula (Hb, containing both the medial and lateral parts), and ventral tegmental area (VTA) according to the Paxinos Altlas (10). Tissue was placed in TRIzol (QIAGEN) and homogenized using an ULTRA THURRAX homogenizer (IKA, Germany). RNA extraction and RT-PCR was performed for *Per2, Bmal1, Vglut2, Orexin* (11), *Cry1* (F primer: AAGTCATCGTGCGCATTTCA; R primer TCATCATGGTCGTCGGACAGA), and *pre-pro-enkephalin*(F primer: CTTGTCAGAGACAGAACGGGT; R primer CCTTGCAGGTCTCCCAGATTT) as described previously (12).Reference genes: Cyclophilin (F primer ATGTGGTCTTTGGGAAGGTG; R primer GAAGGAATGGTTTGATGGGT), β-Actin (F primer ACAACCTTCTTGCAGCTCCTC; R primer CTGACCCATACCCACCATCAC).

### Statistics

All results are expressed as mean ± SEM. Statistical analysis was performed using Graphpad Prism. *T*-tests were performed for two group measures. Two-way ANOVA was performed to detect effects of diet, time or diet, and time interaction on gene expression. When detecting an interaction effect, a Tukey’s HSD *post hoc* test was performed. Results were considered statistically significant at *p* < 0.05.

## Results

During all 6 weeks of the experiment, fcHFHS-fed rats were hyperphagic, cumulatively consuming 3,884 ± 56.23 kcal, compared with 3,041 ± 50.39 kcal ingested by the chow group [*t*_(26)_ = 11.16, *p* < 0.001]. Chow intake in the control group, and chow, fat, and sugar intake in the fcHFHS diet group were significantly higher at night compared with day (Table 1). At the end of the experiment, fcHFHS-fed rats were heavier and more obese than chow-fed rats [BW: 411.3 ± 4.2 vs. 429.7 ± 4.9 g; *t*_(26)_ = 2.38, *p* < 0.001; WAT: 5.6 ± 0.2 vs. 9.9 ± 0.5 g; *t*_(26)_ = 8.34, *p* < 0.001].

**Table 1:** Eating patterns from chow-fed and free-choice high-fat high-sugar (fcHFHS) diet fed groups and mRNA expression from clock-genes *Cry1, Bmal1, Per2*, and the *Vglut2* gene.

Eating patterns								
	**Chow**		**fcHFHS**					
	**Chow**		**Chow**		**Fat**		**Sugar**	
%	100		44.4 ± 1.4		13.5 ± 1.2		41.9 ± 2.2	
Day/night feeding	**Day**	**Night**	**Day**	**Night**	**Day**	**Night**	**Day**	**Night**
			
	16.2 ± 1.1	83.8 ± 1.1	15.7 ± 1.0	84.3 ± 1.0	8.4 ± 1.2	91.5 ± 1.2	22.1 ± 1.0	77.9 ± 1.1

Two-way ANOVA			Time *F*_(1,78)_ = 5,894, *p* < 0.001, diet component *F*_(2,78)_ = 0, *p* > 0.99, interaction *F*_(2,78)_ = 76.6, *p* < 0.001					

*T*-test day vs. night	*t*_(26)_ = 44.9, *p* < 0.001		*t*_(26)_ = 49.3, *p* < 0.001		*t*_(26)_ = 48.1, *p* < 0.001		*t*_(26)_ = 36.2, *p* < 0.001	

**Gene expression**								

**Gene**	**Group**	**Brain area:**	**Suprachiasmatic nucleus**	**Nucleus accumbens**	**Lateral hypothalamus**		**Habenula**	**Ventral tegmental area**

*Cry1*	Chow	Day	4.6 ± 0.3	1.9 ± 0.2	9.3 ± 0.5		5.8 ± 1.5	2.6 ± 0.3
		Night	6.0 ± 0.5	2.4 ± 0.2	11.5 ± 1.4		9.4 ± 2.2	3.2 ± 0.1

	fcHFHS	Day	4.8 ± 0.5	2.2 ± 0.2	8.4 ± 0.5		5.7 ± 0.9	2.6 ± 0.4
		Night	6.5 ± 0.4	2.5 ± 0.2	10.9 ± 0.6		9.9 ± 1.9	2.9 ± 0.2

	Two-way ANOVA	Interaction Diet Time	*F*_(1,23)_ = 0.11; *p* = 0.7 *F*_(1,23)_ = 0.65; *p* = 0.4 ***F*_(1,23)_ = 14.2; *p* < 0.01**	*F*_(1,24)_ = 0.4; *p* = 0.5 *F*_(1,24)_ = 1.0; *p* = 0.3 ***F*_(1,24)_ = 4.4; *p* < 0.05**	*F*_(1,21)_ = 0.06; *p* = 0.7 *F*_(1,21)_ = 0.8; *p* = 0.3 ***F*_(1,21)_ = 9.5; *p* < 0.01**		*F*_(1,23)_ = 0.03; *p* = 0.8 *F*_(1,23)_ = 0.007; *p* = 0.9 ***F*_(1,23)_ = 5.5;*p* < 0.0**5	*F*_(1,24)_ = 0.1; *p* = 0.7 *F*_(1,24)_ = 0.3; *p* = 0.6 *F*_(1,24)_ = 3.4; *p* = 0.07

*Bmal1*	Chow	Day	3.7 ± 0.1	1.9 ± 0.1	9.0 ± 0.6		1.8 ± 0.2	1.5 ± 0.1
		Night	4.2 ± 0.4	1.6 ± 0.1	7.0 ± 0.5		1.7 ± 0.3	1.1 ± 0.1

	fcHFHS	Day	4.4 ± 0.2	1.5 ± 0.3	8.9 ± 0.6		1.9 ± 0.2	1.3 ± 0.2
		Night	3.9 ± 0.2	1.7 ± 0.1	6.6 ± 0.4		1.8 ± 0.3	1.1 ± 0.04

	Two-way ANOVA	Interaction Diet Time	*F*_(1,24)_ = 2.53; *p* = 0.1 *F*_(1,24)_ = 0.7; *p* = 0.4 *F*_(1,24)_ = 0.006; *p* = 0.9	*F*_(1,22)_ = 2.47; *p* = 0.1 *F*_(1,22)_ = 1.4; *p* = 0.2 *F*_(1,22)_ = 0.4; *p* = 0.4	*F*_(1,22)_ = 0.08; *p* = 0.7 *F*_(1,22)_ = 0.3; *p* = 0.5 ***F*_(1,22)_ = 16.5; *p* < 0.01**		*F*_(1,23)_ = 0.001; *p* = 0.9 *F*_(1,23)_ = 0.19; *p* = 0.6 *F*_(1,23)_ = 0.03; *p* = 0.8	*F*_(1,24)_ = 0.7; *p* = 0.3 *F*_(1,24)_ = 1.1; *p* = 0.2 ***F*_(1,24)_ = 9.1; *p* < 0.01**

*Per2*	Chow	Day	2.3 ± 0.2	0.7 ± 0.05	2.4 ± 0.1		0.5 ± 0.1	0.6 ± 0.07
		Night	3.3 ± 0.4	1.2 ± 0.1	4.0 ± 0.4		1.2 ± 0.1	0.9 ± 0.1

	fcHFHS	Day	3.0 ± 0.2	0.7 ± 0.06	2.6 ± 0.2		0.4 ± 0.06	0.6 ± 0.07
		Night	3.3 ± 0.3	0.8 ± 0.05	3.1 ± 0.2		1.2 ± 0.2	1.0 ± 0.08

	Two-way ANOVA	Interaction Diet Time	*F*_(1,24)_ = 1.1; *p* = 0.29 *F*_(1,24)_ = 1.0; *p* = 0.3 *F*_(1,24)_ = 3.4; *p* = 0.07	***F*_(1,22)_ = 5.0; *p* < 0.03** *F*_(1,22)_ = 2.6; *p* = 0.12 ***F*_(1,22)_ = 11.7; *p* < 0.01**	***F*_(1,22)_ = 4.3; *p* < 0.04** *F*_(1,22)_ = 1.1; *p* = 0.2 ***F*_(1,22)_ = 14.2; *p* < 0.01**		*F*_(1,23)_ = 0.08; *p* = 0.7 *F*_(1,23)_ = 0.05; *p* = 0.8 ***F*_(1,23)_ = 14.9; *p* < 0.01**	*F*_(1,24)_ = 0.9; *p* = 0.3 *F*_(1,24)_ = 0.1; *p* = 0.6 ***F*_(1,24)_ = 12.5; *p* < 0.01**

*Vglut2*	Chow	Day	2.2 ± 0.5	0.1 ± 0.02	21.6 ± 1.1		14.7 ± 2.5	11.6 ± 1.4
		Night	2.9 ± 0.6	0.1 ± 0.03	26.8 ± 1.5		22.3 ± 3.7	10.5 ± 0.8

	fcHFHS	Day	1.9 ± 0.2	0.06 ± 0.01	26.9 ± 2.7		16.6 ± 1.6	10.6 ± 1.6
		Night	2.1 ± 0.4	0.07 ± 0.01	24.2 ± 1.4		19.0 ± 3.7	11.7 ± 0.6

	Two-way ANOVA	Interaction Diet Time	*F*_(1,24)_ = 0.31; *p* = 0.5 *F*_(1,24)_ = 0.9; *p* = 0.3 *F*_(1,24)_ = 0.8; *p* = 0.3	*F*_(1,23)_ = 0.22; *p* = 0.6 ***F*_(1,23)_ = 7.16; *p* < 0.05** *F*_(1,23)_ = 0.005; *p* = 0.9	***F*_(1,22)_ = 4.38;*p* < 0.05** *F*_(1,22)_ = 0.56; *p* = 0.4 *F*_(1,22)_ = 0.45; *p* = 0.5		*F*_(1,23)_ = 0.76; *p* = 0.3 *F*_(1,23)_ = 0.06; *p* = 0.8 *F*_(1,23)_ = 2.7; *p* = 0.1	*F*_(1,22)_ = 0.83; *p* = 0.3 *F*_(1,22)_ = 0.0; *p* = 0.9 *F*_(1,22)_ = 0.0; *p* = 0.9

*The upper part of the table shows feeding day–night patterns of chow and fcHFHS rats. Results of the two-way ANOVA comparing daytime (day, nigh) vs. diet component (chow, sugar, fat) on the percentage of caloric intake are shown for the fcHFHS group. Results from the *t*-test analysis of day–night intake are shown under every diet component for chow and fcHFHS groups. In the lower part of the table, the different brain areas are shown in columns. In rows are presented: the studied gene; the diet condition: chow/fcHFHS and; and daytime: day/night. Results from the two-way ANOVA analysis (diet condition vs. daytime) per brain area are shown under every gene description. Significant statistical effects are highlighted in bold letters. Data are presented as mean ± SEM*.

**Figure 1:**
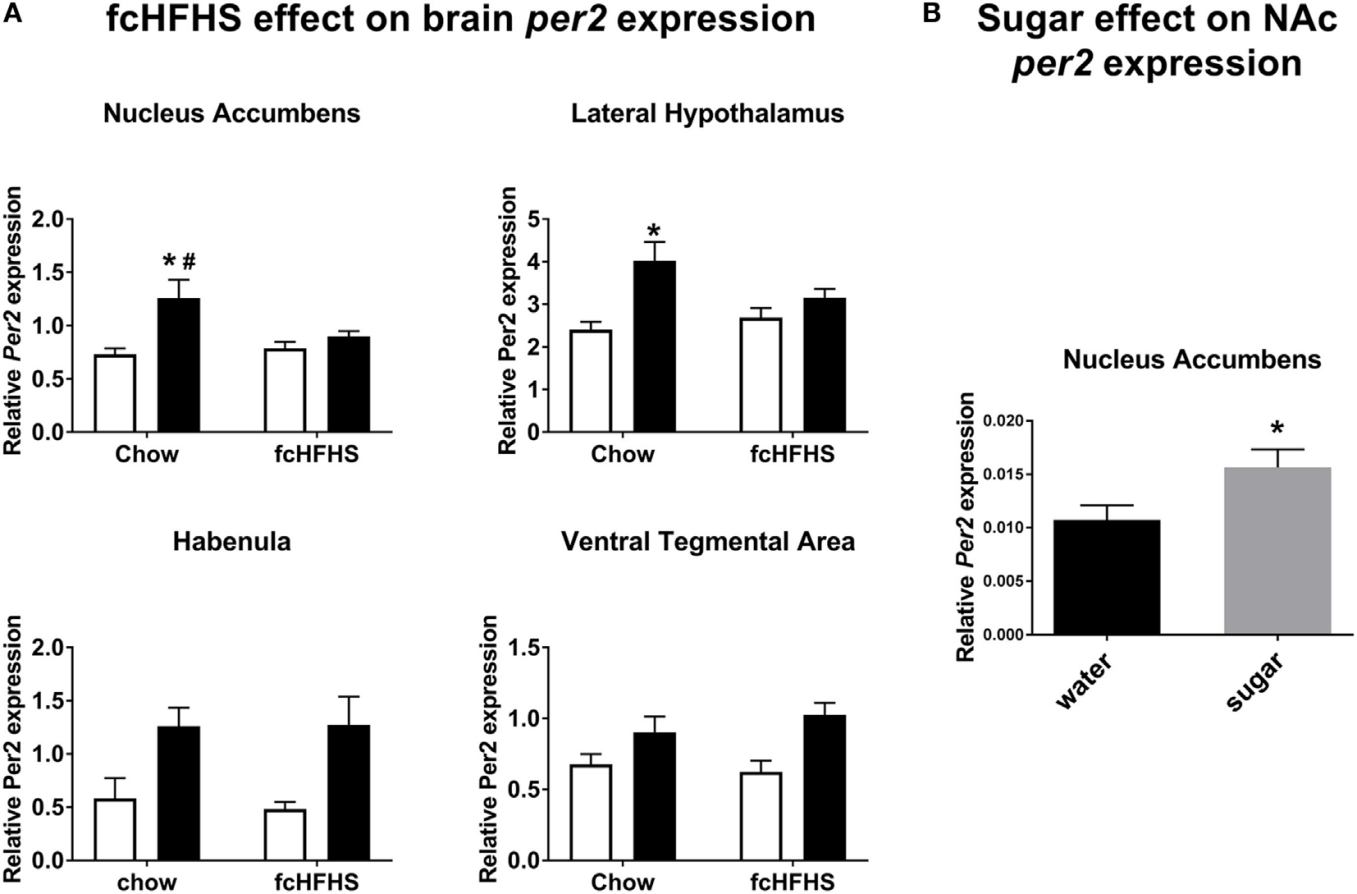
*Per2* mRNA expression in nucleus accumbens (NAc) and lateral hypothalamus, but not habenula or ventral tegmental area, is altered by free-choice high-fat high-sugar (fcHFHS) diet exposure. **(A)** Day (white bars) night (black bars; time factor) expression of *Per2* in chow diet vs. fcHFHS diet groups (diet factor). All the structures showed significant day–night variations, and when an interaction was observed. * indicates a significant day–night difference of *Per2* expression; and ^#^ indicates a significant effect of diet (chow vs. fcHFHS) on *Per2* expression at night. **(B)** *Per2* mRNA expression in the NAc is significantly higher after sugar drinking compared with water drinking in chow-fed rats. * indicates a significant difference in *Per2* expression after water intake vs. sugar intake. Data are presented as mean ± SEM.

In all brain areas from the chow-fed group, *Per2* mRNA was higher at ZT16 (night) than at ZT4 (day). In fcHFHS-fed rats, however, this day–night difference was absent in the NAc and LH; i.e., no significant difference between day and night in animals fed the fcHFHS diet (Figure 1A). *Cry1* and *Bmal1* expression also showed significant day/night differences in most brain areas investigated (Table 1). Interestingly, the loss of day–night differences in the fcHFHS group was restricted to *Per2* (Table 1). We also measured day–night expression of *Vglut2* in all areas, *orexin* in the LH and *pre-pro-enkephalin* in NAc to investigate whether the observed changes in *Per2* were reflected in feeding-regulating genes. No significant changes were observed for *Orexin* [ANOVA: diet *F*_(1,22)_ = 2.83, *p* = 0.1; time *F*_(1,22)_ = 0.008, *p* = 0.9; Int. *F*_(1,22)_ = 0.04, *p* = 0.8], but *Vglut2* was altered in the LH and NAc of the fcHFHS-fed group (Table 1). In the LH, we observed an interaction effect; however, the *post hoc* analysis did not detect differences between night and day in the chow or in the fcHFHS group. In the NAc, *Vglut* mRNA was significantly lower at both day and night in fcHFHS diet-fed rats compared with chow-fed rats (Table 1). *Pre-pro-enkephalin* expression was higher during the light period in both chow-fed (0.052 ± 0.002) and fcHFHS-fed (0.057 ± 0.002) groups compared with the dark period [chow 0.044 ± 0.003; fcHFHS 0.042 ± 0.002; time *F*_(1,22)_ = 27.0, *p* < 0.001], but no significant diet or interaction effects were observed [diet *F*_(1,22)_ = 0.25, *p* = 0.61; interaction *F*_(1,22)_ = 1.6, *p* = 0.2].

Next, we determined the direct effect of sugar intake on *Per2* mRNA expression in the NAc and observed that *Per2* mRNA was significantly increased by sugar ingestion (4.7 ± 0.1 kcal) compared with drinking water (Figure 1B).

## Discussion

We show that the fcHFHS diet produced a specific disruption in day–night *Per2* expression in the NAc and LH, which was not observed for *Cry1* and *Bmal1* mRNA expression. In the LH and NAc, *Per2* mRNA disruption caused by the fcHFHS diet exposure coincided with alterations of *Vglut2* mRNA (Table 1), a marker of glutamatergic activity and excitatory neuronal functions (13), suggesting a relation between the loss of daily *Per2* variation when consuming a fcHFHS diet and changes in neuronal activity. In none of the brain areas studied, we observed a day–night difference in *Vglut2* expression. This could be due to the timing of sampling, missing the trough or peak, or to the neuronal heterogeneity in the studied areas. However, we did observe a clear overall diet effect on *Vglut2* mRNA in the NAc at both time points measured. Given the importance of glutamate in the NAc for dopamine signaling and the previously reported effects of high energy diets on dopamine receptor binding (14), it might be that this reflects a dampening of neuronal activity of NAc dopamine neurons.

The changes in *Per2* mRNA expression, without changes in *Bmal1* and *Cry* mRNA in the NAc and LH of rats fed fcHFHS diet in this study are similar to previous results described in mice where the fcHFHS diet produced changes only in *PER2* but not in BMAL1 protein expression in the LHb (9). Also, after chronic alcohol intake in mice, a specific *Per2* mRNA acrophase shift was observed in the liver while *Cry* and *clock* remained unaffected (15). *In vitro*, the period length and acrophase of *Per2* mRNA expression in cultured hypothalamic neuronal cells are altered after glucose enrichment to the media, whereas *Bmal1* rhythmicity remained unaffected (16). The specific alteration of *Per2* could indicate that this gene is more sensitive than other clock genes to changes in the physiological state (e.g., hypercaloric feeding or chronic alcohol intake), as for instance, the ablation of dopaminergic cells of the VTA decrease *Per2* mRNA expression as well as its protein product (17) which could reflect a direct response to the microenvironment independent of a clock mechanism. On the other hand, it remains to be determined whether this specific *Per2* alteration might be due to an intra-cellular clock-gene de-synchronization that could be reflecting an aberrant clock function.

We also showed that acute sugar consumption when given at the end of the light period increased *Per2* mRNA expression in the NAc. Interestingly, mice with *ad libitum* access to a 5 and 10% sugared water solution consume it mainly during the night phase and this did not disturb *Per2* gene expression in the NAc (18). Taken together, these data suggest that time of sugar intake is an important factor to produce *Per2* alterations in the NAc and that intake at the “wrong” time disturbs the day–night expression of this clock gene. Furthermore, we observed in this study that rats with chronic access to the fcHFHS diet exhibited reduced *Per2* expression in the NAc and LH at night compared with the chow-fed rats. This could indicate that sugar ingestion, in behaviorally rhythmic animals, has to be accompanied with fat ingestion to produce the *Per2* reduction in NAc at night since in the experiment of Bainier et al. (18), where mice ingested only sugar (mainly during the night) *Per2* mRNA expression was similar compared with animals ingesting water. When chronically exposed to the fcHFHS diet which combines sugar and fat, also metabolic changes appear, including high basal blood glucose (19), thus it might be that this prolonged hyperglycemia impacts cell functioning and consequently, produces a clock-gene disruption in the NAc and LH, two areas with no self-sustained oscillations, in which normal rhythmicity could be overridden by abnormal physiological factors such as hyperglycemia. In line with such direct effects of glucose, the NAc and LH contain glucose-sensitive cells (20, 21).

Although we clearly show effects of the fcHFHS diet on *Per2* mRNA in NAc and LH, these changes were not accompanied by changes in feeding rhythm or expression of genes involved in feeding behavior. For example, *pre-pro-enkephalin* mRNA in chow-fed animals showed a clear difference between ZT4 and ZT16, but this was not affected by fcHFHS-diet feeding. Apparently, the changes in *Per2* alone in these areas are not sufficient to induce changes in the daily feeding pattern. Of note, an overall *Per2* mutation in mice does result in loss of the daily rhythm in sucrose drinking (18), pointing to a role for *Per2* in other areas of the brain (or body), or to developmental effects of *Per2* in feeding behavior.

The LH has direct glutamatergic projections to the LHb (22), which could have predicted changes in the Hb as well. We did not find, however, an effect of the fcHFHS diet on rhythmic *Per2* gene expression in the Hb. Possibly light is a stronger *zeitgeber* than food in the Hb, as there are clear light inputs to Hb (23) like is known for the SCN (which also still showed a day/night difference for clock genes). Earlier we showed, in mice, that PER2 protein in the LHb was affected by the fcHFHS diet (9); however, these mice showed clear changes in the daily feeding rhythm of fat and sugar. These results highlight the hierarchical organization of the circadian system; when disturbances are in “weak” brain oscillators (NAc and LH) this does not affect behavior. It remains to be confirmed when a spontaneous change of feeding patterns toward day time does occur in rats, whether this would be accompanied by the same *Per2* disruptions in the LHb as shown for day-snacking mice. We cannot discard that disruptions of *Per2* in NAc and LH could reflect a progressive alteration of the circadian system and with more profound obese state, other areas like LHb would also be compromised.

In this study, the fcHFHS diet did not result in high-fat and/or sugar intake during the light period, as we had previously observed in mice and rats (8, 9). This discrepancy might be due to the amounts of sugar and fat consumed. In previous studies, mice and rats consumed more fat (>30%) than sugar (25%) when fed a fcHFHS diet. In the current experiment, rats consumed only 10% of their total caloric intake as fat, whereas sugar intake was higher than shown before. It is unclear what caused this difference in intake; however, it does point to a role for dietary intake in feeding patterns. Previously we observed that rats, consuming more than 30% fat on the fcHFHS diet, consumed 40% of their sugar intake during the light period (8). Nonetheless, when rats were exposed to only sugar *ad libitum* in addition to chow (fcHS diet), sugar intake was mainly restricted to the dark period (8). The animals in the current experiment drank similar amounts of sugar as animals on the fcHS diet (8), thus, it could well be that although sugar can influence *Per2* in the reward circuitry, this is not sufficient to induce behavioral effects. This points to an additional factor linked to fat feeding that together with altered *Per2* expression mediates disruptions in palatable intake patterns, but only when the total fat intake exceeds a minimum amount. It is clear that sugar intake or fat intake alone does not disrupt behavioral rhythms in rats (8).

Taken together, we show that the fcHFHS diet and acute sugar drinking affect *Per2* gene expression in areas involved in food reward. These *Per2* expression changes, however, were not sufficient to alter feeding-related peptides or feeding behavior.

## Ethics Statement

All experiments were approved by the Animal Ethics Committee of the Royal Netherlands Academy of Arts and Sciences (Amsterdam).

## Author Contributions

AB-V and SF designed experiments. AB-V, UU, LE, and LK performed experiments. AB-V and SF prepared the manuscript. UU, LE, LK, AK, and JM edited the manuscript. SF supervised the entire study.

## Conflict of Interest Statement

The authors declare that the research was conducted in the absence of any commercial or financial relationships that could be construed as a potential conflict of interest.
